# Co-expression patterns of cancer associated fibroblast markers reveal distinct subgroups related to patient survival in oropharyngeal squamous cell carcinoma

**DOI:** 10.3389/fcell.2024.1337361

**Published:** 2024-01-24

**Authors:** Su Ir Lyu, Jannik Johannsen, Adrian Georg Simon, Karl Knipper, Nora Wuerdemann, Shachi Jenny Sharma, Martin Thelen, Kevin Karl Hansen, Caroline Fretter, Charlotte Klasen, Julia Esser, Malte Christian Suchan, Helen Abing, Philipp Heinrich Zimmermann, Anne Maria Schultheis, Hans Anton Schloesser, Jens Peter Klussmann, Alexander Quaas, Hans Nikolaus Caspar Eckel

**Affiliations:** ^1^ Faculty of Medicine and University Hospital of Cologne, Institute of Pathology, University of Cologne, Cologne, Germany; ^2^ Faculty of Medicine and University Hospital of Cologne, Department of Otorhinolaryngology, Head and Neck Surgery, University of Cologne, Cologne, Germany; ^3^ Center for Molecular Medicine Cologne, Faculty of Medicine and University Hospital of Cologne, University of Cologne, Cologne, Germany; ^4^ Faculty of Medicine and University Hospital of Cologne, Department of General, Visceral and Cancer Surgery, University of Cologne, Cologne, Germany; ^5^ Department I of Internal Medicine, Center for Integrated Oncology Aachen Bonn Cologne Duesseldorf, Faculty of Medicine and University Hospital Cologne, University of Cologne, Cologne, Germany

**Keywords:** oropharyngeal cancer, human papillomavirus, head and neck squamous cell carcinoma, platelet-derived growth factor receptor beta, alpha smooth muscle actin, periostin, fibroblast activation protein, cancer associated fibroblasts

## Abstract

**Background:** The incidence of oropharyngeal squamous cell carcinoma (OPSCC) is rapidly increasing in high income countries due to its association with persistent high-risk human papilloma virus (HPV) infection. Recent scientific advances have highlighted the importance of the tumor microenvironment in OPSCC. In this study, including 216 OPSCC patients, we analyze the composition of four established markers of cancer associated fibroblasts (CAFs) in the context of intratumoral CD8 T-cell infiltration.

**Methods:** Immunohistochemical staining for fibroblast activation protein (FAP), platelet-derived growth factor receptor beta (PDGFRb), periostin, alpha smooth muscle actin (α-SMA) and CD8 were analyzed digitally and their association with survival, tumor- and patient characteristics was assessed.

**Results:** Co-expression of CAF markers was frequent but not associated with HPV status. FAP^high^ and PDGFRb^high^ expression were associated with increased CD8 T-cell infiltration. Low expression of PDGFRb improved patient survival in female patients but not in male patients. We identified PDGFRb^low^ periostin^low^ α-SMA^low^ status as an independent predictor of improved survival (hazard ratio 0.377, *p* = 0.006).

**Conclusion:** These findings elucidate the co-expression of four established CAF markers in OPSCC and underscore their association with T-cell infiltration and patient survival. Future analyses of CAF subgroups in OPSCC may enable the development of individualized therapies.

## 1 Introduction

Head and neck squamous cell carcinoma (HNSCC) is the sixth most common cancer entity worldwide. Oropharyngeal squamous cell carcinoma (OPSCC), comprising cancers of the base of tongue, tonsils, uvula, and soft palate, is a subtype of HNSCC distinguished by anatomical location and molecular characteristics. OPSCC, like other HNSCCs, has canonically been associated with alcohol and tobacco consumption as driving factors of carcinogenesis. However, persistent infection with carcinogenic high risk human papillomavirus (HPV) has emerged as an important risk factor of OPSCC in the past decades and the incidence of HPV-positive OPSCC is rising in high-income countries (Klussmann et al., 2003; Lechner et al., 2022). HPV driven OPSCC is characterized by a distinct pathogenesis and improved survival compared to HPV-negative cancers (Kian Ang et al., 2010; Mehanna et al., 2023).

In recent years, immune checkpoint blockade (ICB) has emerged as promising therapy for multiple cancer types including OPSCC (Ferris et al., 2016). As the efficacy of ICB remains limited in OPSCC and other types of solid cancer, they have gathered renewed interest in the tumor microenvironment (TME) (Elmusrati et al., 2021). The TME is comprised of extracellular matrix (ECM) and a variety of infiltrating cells including lymphocytes, tumor associated macrophages, myeloid derived suppressor cells, innate lymphoid cells, fibroblasts, and cancer associated fibroblasts (CAFs) (Pitt et al., 2016; Nakamura and Smyth, 2019; Elmusrati et al., 2021; Mao et al., 2021). CAFs arise from various cell types and are a primary source of ECM modulation, angiogenesis, cell migration and nutrition (Zhang et al., 2022). While some studies demonstrate a protumorigenic role of CAFs in HNSCC including cancer stem cell renewal, immune cell evasion and chemoresistance, evidence from other malignancies suggests a complimentary tumor-suppressive role of CAFs (Özdemir et al., 2014; Brechbuhl et al., 2017; Su et al., 2018; Galbo et al., 2021).

Currently, no definitive classification of CAFs has been established in HNSCC or other cancer types as this scientific field is still rapidly evolving and new insights often challenge the established paradigms of CAF-population defining signatures. (Brechbuhl et al., 2017; Chen et al., 2021; Galbo et al., 2021; Foster et al., 2022; Cords et al., 2023). Fibroblast activation protein (FAP), a serine protease and well-established CAF surface protein, is commonly overexpressed in activated fibroblasts and high expression is associated with worse survival in multiple cancer types (Liu et al., 2015). Targeted therapies directed against FAP including monoclonal antibodies, drug conjugates and FAP directed chimeric antigen receptor (CAR) T cells are currently under investigation (Busek et al., 1933; Ostermann et al., 2008). Furthermore, [68 Ga]Ga-labeled inhibitors of FAP ([68 Ga]Ga-FAPI-46) have been developed for positron emission tomography (PET/CT) imaging utilizing CAFs to improve detection rate of nodal metastasis and optimize radiotherapy planning (Kratochwil et al., 2019; Wegen et al., 2022). α smooth muscle actin (α-SMA) is involved in the contractile apparatus of smooth muscle cells and is commonly used to defined activated myofibroblasts (myCAFs) (Tomasek et al., 2002). myCAFs partake in the reorganization of the extracellular matrix and increase tissue stiffness contributing to impaired chemosensitivity (Shen et al., 2020). Periostin, canonically linked to pulmonary fibrosis and allergy (Sonnenberg-Riethmacher et al., 2021), is an extracellular matrix protein associated with epithelial-mesenchymal transition and tissue invasion and formation of metastases in HNSCC (Kudo et al., 2006). Expression of platelet-derived growth factor receptor beta (PDGFRb) regulates neo angiogenesis and is expressed in various malignancies including HNSCC (Bran et al., 2009).

As fibroblasts surrounding a cancerous lesion can promote tumor growth or act as tumor suppressors, evaluation of prognostic impact and specific identification of relevant targets for personalized therapy is warranted (Kanzaki and Pietras, 2020). Targeting tumor promoting CAFs may enable avenues for targeted therapy leading to the disinhibition of response to conventional therapies and minimize adverse events. In this study we aimed to describe the expression of FAP, PDGFRb, periostin, and α-SMA in a large cohort of HPV-positive and HPV-negative OPSCC patients and elucidate the association with CD8 T-cell infiltration and prognosis.

## 2 Materials and methods

### 2.1 Patient cohort and tumor samples

Patients included in this study were diagnosed with OPSCC [C09, C10, International Classification of Diseases for Oncology (ICD-O)] and treated at the University Hospital Cologne between 2005 and 2020. Patients with sufficient pretherapeutic tissue available were included in this study. Tissue microarrays (TMA) were prepared from formalin-fixed, paraffin-embedded (FFPE) cancer tissue resulting in 216 samples. Clinical and pathological features of the patient cohort were ascertained from medical records and are displayed in Table 1. The study was conducted in accordance with the declaration of Helsinki. The study protocol was approved by the Ethics committee of the University of Cologne (study number 19-1288).

**TABLE 1:** Clinicopathological features of oropharyngeal squamous cell carcinoma patient cohort according to HPV status.

Risk factors		All		HPV-positive		HPV-negative		*p*
		*n* = 216	100 (%)	*n* = 107	49 (%)	*n* = 109	51 (%)	
Nicotine	No nicotine	67	32	49	47	18	17	**<0.001**
	Nicotine abuse	146	68	56	53	90	83	
Alcohol	No alcohol	146	68	88	83	58	53	**<0.001**
	Alcohol abuse	69	32	18	17	51	47	
Age	Young (<60 years)	119	55	60	56	59	54	0.774
	Old (≥60 years)	97	45	47	44	50	46	
Sex	Female	45	21	24	22	21	19	0.567
	Male	171	79	83	78	88	81	
Tumor characteristics								
Localization	Tonsil	149	69	81	76	68	62	**0.002** ^a^
	Bottom of tongue	51	24	24	23	27	25	
	Other	15	7	1	1	14	13	
T-stage	T1-2	120	56	69	64	51	47	**0.009**
	T3-4	96	44	38	36	58	53	
N-stage	N0	46	21	14	13	32	29	**0.003**
	N+	170	79	93	87	77	71	
M-stage	M0	209	97	106	99	103	94	0.119^a^
	M+	7	3	1	1	6	6	
UICC stage	I-III	176	81	103	96	73	67	**<0.001** ^**a**^
	>III	40	19	4	4	36	33	
Grading	G1-2			n.a.		70	79	n.a.
	G3-4					19	21	
Treatment								
Upfront surgery	Yes	132	61	71	66	61	56	0.117
	No	84	39	36	34	48	44	

*p*-values calculated by X^2^ test (Pearson, asymptotic, two-sided) or Fisher’s two-sided exact test (marked by a). Significant *p*-values (*p* ≤ 0.05) in bold.

Disease extent was defined according to UICC TNM 7th or 8th edition according to the valid classification at the time of diagnosis. Based on established TNM status, tumor stage was defined according to the American Cancer Staging Classification 8 (AJCC8 I-IV) for all patients.

### 2.2 p16INK4a immunohistochemistry and HPV-DNA genotyping

To determine the HPV association of OPSCCs, p16^INK4a^ (p16) immunohistochemistry (IHC) and HPV-DNA genotyping was performed for all patients. p16 expression was determined using the Zytomed histology kit (Zytomed Systems, Berlin, Germany) according to the supplier’s and standard protocols (Klussmann et al., 2003). Extracted DNA was analyzed for the presence of HPV-DNA and HPV genotypes (6, 11, 16, 18, 31, 33, 35, 39, 42, 44, 45, 51, 52, 53, 54, 56, 58, 59, 61, 62, 66, 67, 68, 70, 72, 73, 81, 82, 83, 84, 90, 91) by amplification of highly conserved regions of viral genome (L1 Region, primer pairs MY11/19, and 125′) followed by DNA/DNA hybridization to assess specificity. Cancers were considered HPV-positive when p16 IHC staining was positive on 70% of tumor cells and high-risk HPV DNA of the aforementioned types was detected. Other combinations (HPV DNA^+^/p16^−^, HPV DNA^−^/p16^−^, HPV DNA^−^/p16^+^) were considered HPV negative.

### 2.3 Preparation of tissue microarrays and immunohistochemistry

Sufficient FFPE cancer tissue with a thickness of at least 2 mm was mandatory to produce TMA cores. TMAs were assembled as previously described (Simon, 2008). Briefly, 1.2 mm cores were taken from a tumor area including invasive margins marked by an experienced pathologist. A 1.2 mm cylinder was removed using a semi-automated punch press and transferred to a fresh paraffin block. For staining, 4 µm thick slides were freshly cut from TMAs.

Antibodies targeting α-SMA, PDGFRb, periostin, FAP and CD8 were used for immunohistochemical staining according to the manufacturers recommended protocols. Antibody specifications are displayed in Supplementary Table S1. Automated staining was performed with the Leica BOND-MAX automated system (Leica Biosystems, Wetzlar, Germany) in accordance with the manufacturer’s protocol. Stained slides were scanned with the Aperio GT 450 DX (Leica Biosystems, Wetzlar, Germany). Digitalized slide scans were analyzed using an adapted QuPath v0.3.2 protocol as was described previously (Bankhead et al., 2017). After initial cell classification and identification of the cell stroma, the following parameters were defined to detect α-SMA, PDGFRb, periostin and FAP staining as published previously (Knipper et al., 2023): Threshold with the resolution of 2.11 µm/pixel, channel DAB, prefilter Gaussian, smoothing sigma 1, Threshold 0.05. For FAP, PDGFRb, periostin and α-SMA, the cutoff for high expression was defined as the median of the patient population. Values equal or lower to the median were defined as low. The following parameters were defined for CD8 T-cell detection: Detection image optical density sum, requested pixel size 0.5 µm; and cell parameters; cell expansion 5 μm, cell nucleus included; nucleus parameters; background radius 8 μm, median filter radius 0 μm, sigma 1.5 µm, minimum area 10 µm^2^, maximum area 400 μm^2^; intensity parameters; threshold 0.1, max background intensity 2. For CD8 T-cell infiltration, expression <50 lymphocytes/mm^2^ were defined as low and ≥50 lymphocytes/mm^2^ were defined as high. Human tonsil and appendix tissue on each of the TMA slides served as control for staining.

### 2.4 Statistical analysis

Statistical analyses were performed using SPSS statistical software (IBM SPSS 28.0, Armork, NY, United States) and Graphpad Prism (Graphpad Prism v8, San Diego, CA, United States). Differences in patient characteristics and correlation of fibroblast marker expression and expression of CD8 were calculated using Pearson’s Chi-squares test or Fisher’s exact test as appropriate. In Table 2, correction of false discovery rate (FDR) with a FDR of 5% using the original method of Benjamini-Hochberg was conducted (Benjamini and Hochberg, 1995). This resulted in a threshold for significant *p*-values (*p* ≤ 0.012). Overall survival (OS) was calculated from the initial date of histological diagnosis to date of death or loss to follow-up. Patients were followed for a maximum of 10 years. To plot survival curves, the Kaplan-Meier method was used and significance between groups was determined using the two-sided log-rank test. Cox proportional-hazards models were used to determine hazard ratios (HR) and a confidence interval (CI) of 95% in univariate and multivariate analyses. Upset plots were generated using the ComplexUpset packages in R v3.6.3 and Inkscape v1.0. All tests were two-sided and *p*-values ≤0.05 were considered significant if not specifically stated otherwise.

**TABLE 2:** Relation of fibroblast activation protein (FAP), platelet-derived growth factor receptor beta (PDGFRb), periostin and alpha-smooth muscle actin (α-SMA) expression to each other, wih CD8-positive TILs and clinical characteristics (*n* = 216).

		FAP					PDGFRb					Periostin					α-SMA				
		High	%	Low	%	*p*	High	%	Low	%	*p*	High	%	Low	%	*p*	High	%	Low	%	*p*
Expression		105	50	106	50		106	50	106	50		105	50	106	50		106	50	107	50	
FAP	High						55	53	48	47	0.297	66	63	38	37	**<0.001**	56	54	48	46	0.270
	Low						48	46	56	54		38	36	68	64		49	46	57	54	
PDGFRb	High	55	53	48	47	0.297						62	60	41	40	**0.003**	61	59	43	41	**0.010**
	Low	48	46	56	54							41	39	63	61		43	41	62	59	
Periostin	High	66	64	38	36	**<0.001**	62	60	41	40	**0.003**						62	59	43	41	**0.009**
	Low	38	36	68	64		41	39	63	61							43	41	62	59	
α-SMA	High	56	53	49	47	0.270	61	59	43	41	**0.010**	62	59	43	41	**0.009**					
	Low	48	46	57	54		43	41	62	59		43	41	62	59						
CD8	High	65	56	50	44	0.032	67	59	46	41	**0.003**	57	50	57	50	0.941	52	45	63	55	0.15
	Low	40	42	56	58		38	39	60	61		48	49	49	51		54	55	44	45	
Clinical Parameter																					
UICC-Stage	I-III	83	48	90	52	0.268	85	49	87	51	0.726	85	49	88	51	0.696	86	49	89	51	0.697
	IV	22	58	16	42		21	53	19	47		20	53	18	47		20	53	18	47	
Nicotine	f/c	72	50	71	50	0.839	73	50	73	50	0.840	73	51	70	49	0.678	76	52	69	48	0.492
	Never	32	49	33	51		31	49	32	51		30	46	35	54		29	45	36	55	
Alcohol	Abuse	32	47	36	53	0.511	37	54	31	46	0.427	37	54	31	46	0.372	38	55	31	45	0.359
	No alcohol	73	51	69	49		69	48	74	52		67	47	75	53		68	48	75	52	
Sex	Female	24	56	19	44	0.374	23	54	20	46	0.608	26	62	16	38	0.790	22	51	21	49	0.837
	Male	81	48	87	52		83	49	86	51		79	47	90	53		84	49	86	51	
HPV-Status	Positive	49	46	57	54	0.302	49	48	54	52	0.492	48	46	57	54	0.242	49	46	58	54	0.244
	Negative	56	53	49	47		57	52	52	48		57	54	49	46		57	54	49	46	

*p*-values calculated by X^2^ test (Pearson, asymptotic, two-sided), correction of false discovery rate (FDR) with a FDR of 5% using the original method of Benjamini-Hochberg resulted in a threshold of significant *p*-values (*p* ≤ 0.012), significant *p*-values marked in bold; f/c, former/current.

## 3 Results

### 3.1 Patient and tumor characteristics

We included 107 (49%) HPV-positive and 109 (51%) HPV-negative OPSCC patients for a total of 216 analyzed tumors in this study. All patients were treated in curative intent. Patient characteristics are displayed in Table 1. The cohort was comprised of 171 (79%) male and 45 (21%) female patients and 119 patients (55%) were younger than 60 years old while 97 (45%) were under 60 years of age. In the entire cohort, 68% of patients reported nicotine abuse and 32% abused alcohol previously or at the time of diagnosis (self-reported “frequent” or “daily” consumption was considered abuse), while in HPV-negative patients the misuse of both substances was significantly more frequent (nicotine 83% vs. 53%, *p* < 0.001; alcohol 47% vs. 17%, *p* < 0.001). The most common tumor localization was the tonsil (*n* = 149, 69%), followed by the base of the tongue (*n* = 51, 24%). HPV-positive carcinomas were more frequently located in the tonsils (76% vs. 62%, *p* = 0.002).

Overall, 120 patients (56%) were classified as T1-2, whereas 170 (79%) showed lymphonodal infiltration at initial diagnosis. Seven patients suffered from metastatic disease (3%). In HPV-negative patients, stage four disease was significantly more common compared to HPV-positive patients (UICC stage > III in HPV neg. 33% vs. HPV pos. 4%, *p* < 0.001). HPV-negative tumors were classified as grade (G) 1-2 in 70 (79%) cases while 19 (21%) tumors were high grade (G 3-4). Grading was not applied for HPV-positive tumors (Pignon et al., 2009; Ferris and Westra, 2023). For curative treatment, 132 patients (61%) underwent upfront surgery while 84 (39%) of patients received primary (chemo-) radiation.

### 3.2 Distribution of cancer-associated fibroblast marker expression

**FIGURE 1:**
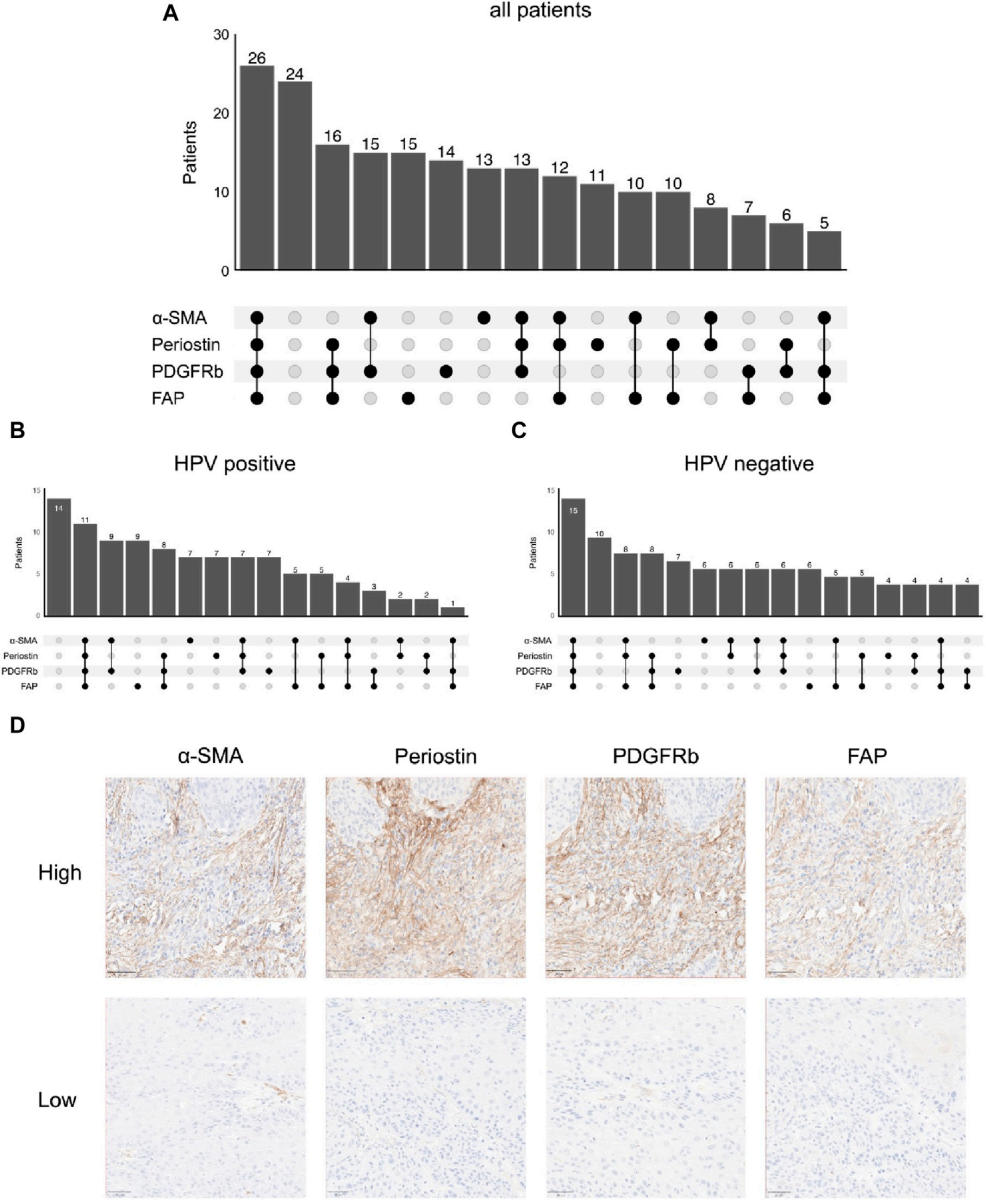
Co-expression patterns of fibroblast markers in immunohistochemistry; number of patients with high/low stromal expression of different combinations of α-smooth muscle actin (α-SMA), periostin, platelet derived growth factor receptor beta (PDGFRb) and fibroblast activation protein (FAP) in **(A)** all patients (*n* = 207), **(B)** human papilloma virus (HPV)-positive patients (*n* = 101) and **(C)** HPV-negative patients (*n* = 104), **(D)** Representative images of immunohistochemical stainings (top high, bottom low) for fibroblast activation protein (FAP), platelet derived growth factor receptor beta (PDGFRb), periostin and α-smooth muscle actin (α-SMA).

As fibroblasts are an essential component of the TME, we performed immunohistochemical staining of FAP, α-SMA, PDGFRb and periostin. Following digital analysis, we divided the patient cohort according to median expression in tumor stroma (FAP: *n* = 106 low, *n* = 105 high; PDGFRb: *n* = 106 low, *n* = 106 high; periostin: *n* = 106 low, *n* = 105 high; α-SMA: *n* = 107 low, *n* = 106 high) (Table 2). Notably, expression of periostin was significantly associated with all other CAF markers (periostin-FAP *p* < 0.001; periostin-PDGFRb *p* = 0.003; periostin-α-SMA *p* = 0.009) while α-SMA was associated with periostin and PDGFRb (*p* = 0.01). Co-expression of fibroblast markers was common as 26 tumors had high expression of all four molecules while 24 were classified low for all markers (Figure 1A). The third most common expression pattern present in 16 patients was periostin^high^ PDGFRb^high^ FAP^high^ α-SMA^low^. Tumors with low expression of all four fibroblast activation markers were the largest population in HPV-positive tumors while combined high expression of all four markers was the most frequent pattern in HPV-negative OPSCC (Figures 1B–D). To elucidate the connection of fibroblast frequency with T cell infiltration in the TME, we measured CD8 T-cell infiltration. Considering a threshold of >50 CD8 T lymphocytes/mm^2^ as high, 117 (54%) tumors were highly infiltrated by CD8 T-cells while 98 (46%) were considered low. CD8 T-cell infiltration was positively associated PDGFRb (*p* = 0.003) high tumors. Expression of FAP, α-SMA, PDGFRb or periostin was independent from nicotine or alcohol abuse, UICC stage, sex or HPV status.

### 3.3 Survival analysis

**FIGURE 2:**
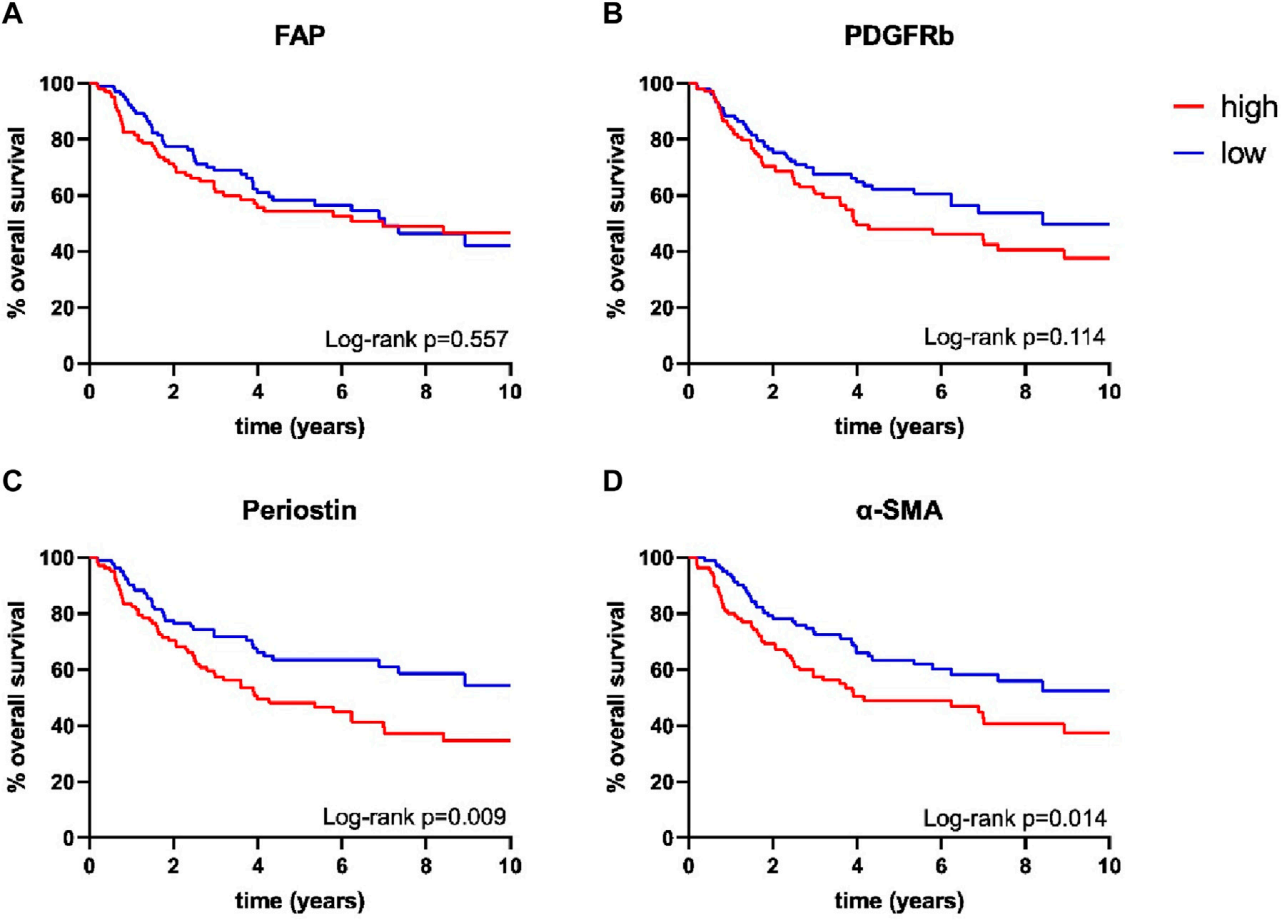
Kaplan-Meier curves for overall survival according to **(A)** fibroblast activation protein (FAP) (high *n* = 105, low *n* = 106), **(B)** platelet derived growth factor receptor beta (PDGFRb) (high *n* = 106, low *n* = 106), **(C)** periostin (high *n* = 105, low *n* = 106) and **(D)** α-smooth muscle actin (α-SMA) (high *n* = 106, low *n* = 107) expression; *p*-values calculated by log-rank test.

**FIGURE 3:**
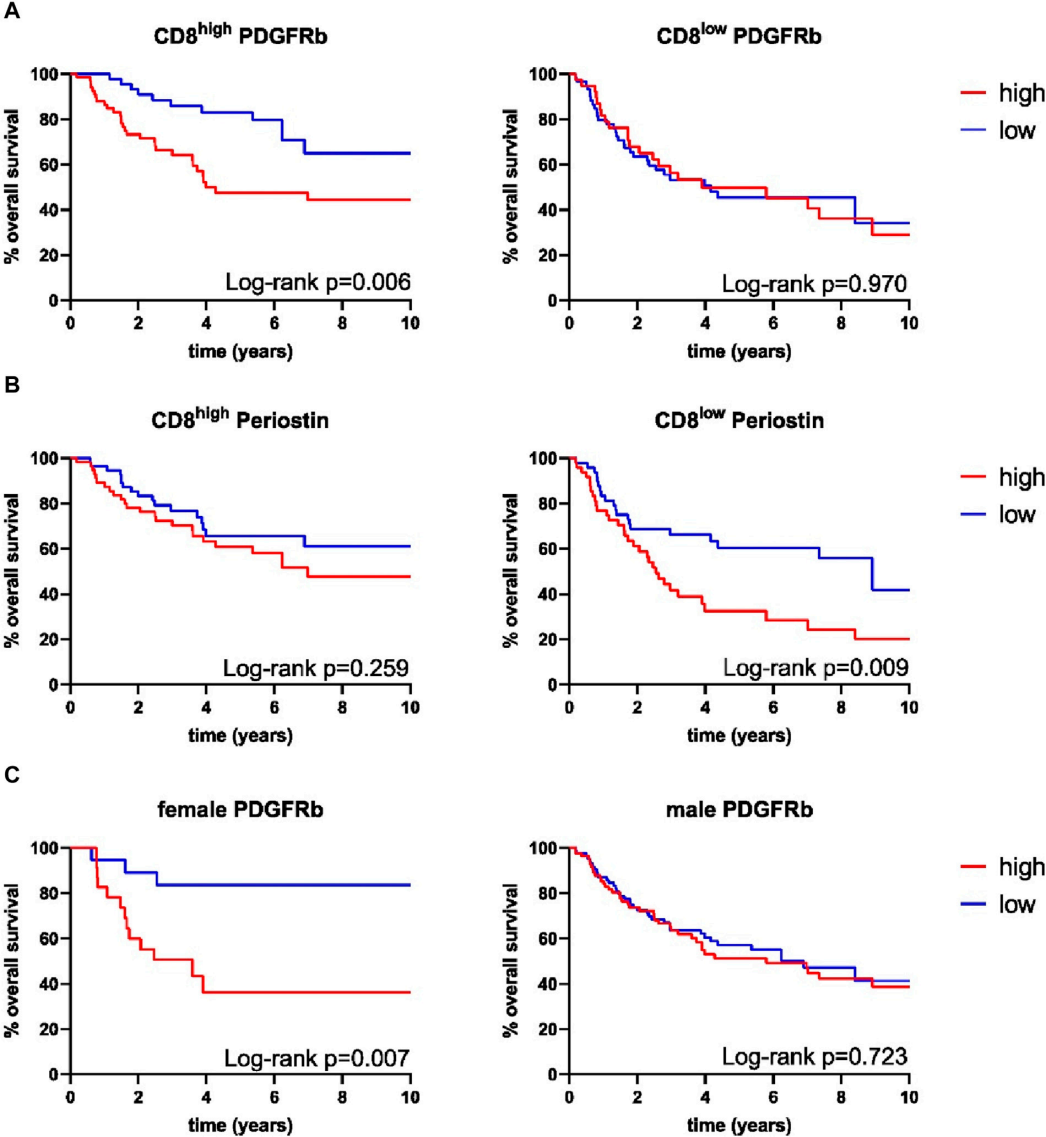
Kaplan-Meier curves for overall survival of combined expression of **(A)** platelet derived growth factor receptor beta (PDGFRb) in CD8^high^ samples (high *n* = 67, low *n* = 46), PDGFRb in CD8^low^ samples (high *n* = 38, low *n* = 60), **(B)** periostin in CD8^high^ samples (high *n* = 38, low *n* = 60), periostin in CD8^low^ samples (high *n* = 48, low *n* = 49), **(C)** PDGFRb in female samples (high *n* = 23, low *n* = 20), PDGFRb in male samples (high *n* = 83, low *n* = 86), *p*-values calculated by log-rank test.

Survival analysis was performed using the Log-Rank test as well as univariate and multivariate Cox regression to determine the association of CAFs and patient survival. Patient follow-up was conducted for a maximum of 10 years; mean follow up for survivors was 66 months. Periostin (*p* = 0.009) and α-SMA (*p* = 0.014) expression were associated with worse overall survival in univariate analyses (Figure 2). FAP (*p* = 0.557) and PDGFRb (*p* = 0.114) expression were not associated with OPSCC patient survival. High CD8 T-cell abundance was correlated to improved patient survival (*p* = 0.004) (Tables 3, 4). Stratifying patients according to CD8 T-cell infiltration revealed high PDGFRb expression as a predictor of impaired survival in the CD8^high^ population (*p* = 0.006) (Figure 3A). PDGFRb status did not impact survival in the CD8^low^ group (*p* = 0.970). In contrast, high periostin expression was associated with worse overall survival in the CD8^low^ group (*p* = 0.009) while no impact on patient survival was observed in the CD8^high^ group (*p* = 0.259) (Figure 3B). In the subgroup of HPV-positive tumors, individual expression of CAF markers did not reveal a significant association with overall survival (Supplementary Figure S2). Canonically, most OPSCC cases occur in men and the investigation of sex specific aspects and distinct molecular patterns in female patients have only recently been appreciated (Tosi et al., 2022). To elucidate the association of fibroblast activation markers with sex specific patient survival, we performed subgroup analysis by sex. Notably, PDGFRb^high^ expression status was not associated with patient survival in men but in female patients, PDGFRb^high^ status conferred poor overall survival (*p* = 0.007) (Figure 3C).

**TABLE 3:** Univariate and multivariate survival analysis according to tumor characteristics and risk factors (*n* = 216).

Univariate												Multivariate		
Median survival (Months)														
Variable		*n*	OS	CI		*p*	HR	CI		*p* ^*a*^	HR	CI		*p* ^*a*^
				Lower	Upper			Lower	Upper			Lower	Upper	
FAP	High	105	n.a.	n.a.	n.a.	0.557	0.885	0.588	1.334	0.56	0.961	0.622	1.485	0.857
	Low	106	84	48.568	119.432									
PDGFRb	High	106	47	8.483	85.517	0.114	0.722	0.48	1.086	0.118	1.153	0.746	1.782	0.522
	Low	106	n.a.	n.a.	n.a.									
Periostin	High	105	47	21.467	72.533	**0.009**	1.725	1.138	2.613	**0.010**	1.488	0.943	2.347	0.088
	Low	106	n.a.	n.a.	n.a.									
α-SMA	High	106	49	9.247	88.753	**0.014**	1.665	1.102	2.517	**0.015**	1.406	0.915	2.16	0.120
	Low	107	n.a.	n.a.	n.a.									
CD8	High	117	n.a.	n.a.	n.a.	**0.004**	0.555	0.369	0.837	**0.005**	0.643	0.421	0.983	**0.042**
	Low	98	49	10.798	87.202									
HPV-status	Positive	107	n.a.	n.a.	n.a.	**<0.001**	0.329	0.212	0.51	**<0.001**	0.528	0.324	0.858	**0.010**
	Negative	109	35	23.824	46.176									
UICC-Stage	IV	40	16	5.113	26.88	**<0.001**	3.988	2.527	6.293	**<0.001**	2.962	1.785	4.915	**<0.001**
	I-III	176	n.a.	n.a.	n.a.									
Sex	Female	45	n.a.	n.a.	n.a.	0.324	0.765	0.447	1.31	0.329	0.800	0.462	1.385	0.425
	Male	171	82	44.888	119.112									

HR hazard ratio estimated by COX proportional-hazards models; CI 95% confidence interval; n.a., not applicable; *p*-values calculated by Log Rank (Mantel-Cox) test; univariate; (p^a^ -values) estimated by Cox proportional-hazards model, uni- and multivariate analyses; *p*-values (*p* ≤ 0.05) in bold; HR, hazard ratio; OS, overall survival, given in months; CI 95%, confidence interval.

**TABLE 4:** Univariate and multivariate survival analysis according to tumor characteristics and combined expression of platelet-derived growth factor receptor beta (PDGFRb), periostin and α-smooth muscle actin (α-SMA).

Univariate												Multivariate		
Median survival (Months)														
Variable				CI				CI				CI		*P* ^***b***^
		*n*	OS			*p* ^*a*^	HR			*P* ^*b*^	HR			
				Lower	Upper			Lower	Upper			Lower	Upper	
PDGFRb α-SMA Periostin	Low	39	n.a.	n.a.	n.a.	**0.003**	0.362	0.182	0.72	**0.004**	0.377	0.189	0.752	**0.006**
	SH, DH, TH	167	64	37.178	90.822									
CD8 Expression	High	117	n.a.	n.a.	n.a.	**0.004**	0.555	0.369	0.837	**0.005**	0.627	0.414	0.948	**0.027**
	Low	98	49	10.798	87.202									
HPV-Status	Positive	107	n.a.	n.a.	n.a.	**<0.001**	0.329	0.212	0.51	**<0.001**	0.497	0.307	0.804	**0.004**
	Negative	109	35	23.824	46.176									
UICC-Stage	IV	40	16	5.113	26.88	**<0.001**	3.988	2.527	6.293	**<0.001**	2.806	1.696	4.644	**<0.001**
	I-III	176	n.a.	n.a.	n.a.									
Sex	Female	45	n.a.	n.a.	n.a.	0.324	0.765	0.447	1.31	0.329	0.847	0.494	1.454	0.547
	Male	171	82	44.888	119.112									

(p^a^ -values) calculated by Log Rank (Mantel-Cox) test; univariate; (p^b^ -values) estimated by Cox proportional-hazards models, uni- and multivariate analyses; *p*-values (*p* ≤ 0.05) in bold; SH, single high; DH, double high; TH triple high; n.a., not applicable; HR, hazard ratio; OS, overall survival, given in months; CI 95%, confidence interval.

In survival analysis by multivariate Cox regression, positive HPV-status was associated with improved survival in univariate and multivariate analyses (HR 0.528, CI 0.324–0.858, *p* = 0.01) (Table 1). Tumor infiltration of >50 CD8 T lymphocytes/mm^2^ improved patient survival significantly (HR 0.643, CI 0.421–0.983, *p* = 0.042) while a higher UICC stage (Stage I-III vs. Stage IV) was associated with worse survival (HR 2.962, CI 1.785–4.915, *p* < 0.001). FAP, PDGFRb, periostin and α-SMA as individual parameters did not predict patient survival (FAP *p* = 0.857, PDGFRb *p* = 0.522, periostin *p* = 0.088, α-SMA *p* = 0.120) in multivariate COX regression (Table 3).

**FIGURE 4:**
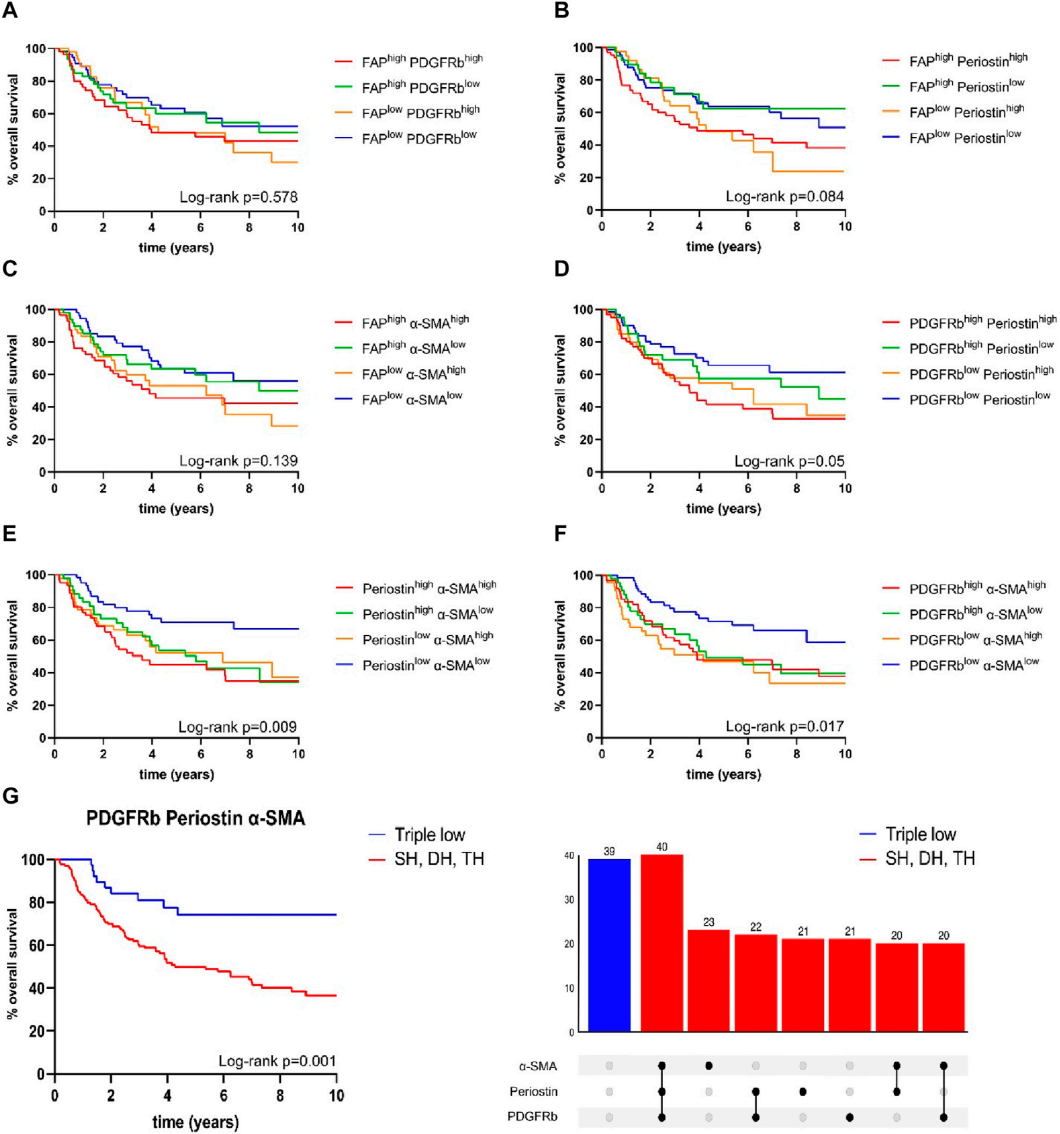
Kaplan-Meier curves for overall survival in correlation to expression status of **(A)** fibroblast activation protein (FAP)^high/low^ platelet derived growth factor receptor beta (PDGFRb)^high/low^, **(B)** FAP^high/low^ periostin^high/low^, **(C)** FAP^high/low^ α-smooth muscle actin (α-SMA)^high/low^, **(D)** PDGFRb^high/low^ periostin^high/low^, **(E)** periostin^high/low^α-SMA^high/low^, **(F)** PDGFRb^high/low^α-SMA^high/low^; **(G)** PDGFRb^low^, α-smooth muscle actin (α-SMA)^low^, periostin^low^ samples (*n* = 39) compared to samples high for one or more markers (*n* = 167); *p*-values calculated by log-rank test; SH single high, DH double high, TH triple high, *p*-values calculated by log-rank test.

To allow the further characterization of fibroblast activation marker expression patterns, we analyzed co-expression of the four examined fibroblast activation markers and assessed their correlation with patient survival (Figure 4). Expression patterns of FAP^high/low^ periostin^high/low^ (*p* = 0.084), FAP^high/low^ α-SMA^high/low^ (*p* = 0.139) and FAP^high/low^ PDGFRb^high/low^ (*p* = 0.578) were not associated with improved patient survival (Figures 4A–C). The combined expression status of PDGFRb^high/low^ periostin^high/low^ just reached a statistically significant impact on overall survival (*p* = 0.05) (Figure 4D). Combined status of periostin^high/low^ α-SMA^high/low^ (*p* = 0.009) and PDGFRb^high/low^ α-SMA^high/low^ (*p* = 0.017) facilitated the stratification of patient survival as for both combinations of CAF markers, double low expression status had the highest overall survival. As co-expression of other CAF markers with FAP did not reveal significant association with patient survival, we focused on the combined expression status of PDGFRb, α-SMA and periostin in relation to patient survival. PDGFRb^low^ α-SMA^low^ periostin^low^ status was associated with improved overall survival in comparison to patients with high expression of one, two or three markers in univariate and multivariate survival analysis (HR 0.377, CI 0.189–0.752, *p* = 0.006) (Figure 4G) (Table 4).

## 4 Discussion

The heterogenous population of CAFs in OPSCC remains unsatisfactorily defined. However, our data clearly show an association of FAP and PDGFRb expression with intratumoral CD8 T-cell infiltration and we identify a PDGFRb^low^ α-SMA^low^ periostin^low^ expression pattern as an independent predictor of improved survival in OPSCC. Therefore, the composition of intratumoral and peritumoral CAFs may be a biomarker to identify suitable patients for future treatment de-escalation trials. Targeting CAFs and specific CAF subpopulations, potentially in combination with immunotherapy, is an attractive avenue for the development of future targeted therapies in OPSCC.

Targeted immunotherapies have improved patient survival rates, but less than 20% of patients have achieved durable tumor regression (Ferris et al., 2016; Burtness et al., 2019). To further improve immunotherapy outcome, a comprehensive understanding of the TME, which includes diverse infiltrating cell types including CAFs, is required. Furthermore, PET/CT imaging using [68 Ga]-radiolabeled inhibitors of FAP utilizes CAFs to detect cancer tissue, thus enabling the classification of disease extent and spread based on CAF abundance. The heterogeneity of CAFs and their subtypes complicates the identification of one common marker for activated CAFs that can be established for routine clinical testing and patient stratification. The concept of α-SMA upregulation as a sign for activated CAFs is currently questioned due to existence of α-SMA^low^ CAFs (Patel et al., 2018). With the rise of high-resolution sequencing techniques, including single cell RNA sequencing, multiple gene signatures and biomarkers for CAFs have been identified primarily at the RNA level (Yamamoto et al., 2022; Zhou et al., 2022; Dong et al., 2023; Dwivedi et al., 2023). CAFs are presumed to be of various cell origins with diverse protumorigenic and arguably also tumor suppressive functions (Kalluri, 2016; Wang et al., 2022; Grout et al., 2022). This study investigated expression patterns of the previously established fibroblast markers FAP, α-SMA, PDGFRb and periostin in 216 OPSCC patients to not only be able to define co-expression patterns of CAF markers; but also create a tool that can find its way to routine clinical diagnostics in the future.

Oropharyngeal cancer caused by persistent high-risk HPV infection is a distinct cancer entity with different molecular and clinical features and improved patient survival when compared to HPV-negative OPSCC (Lechner et al., 2022; Mehanna et al., 2023). In line with extensive previously published evidence, HPV positivity conferred improved survival in our patient cohort (Ang et al., 2010; Lechner et al., 2022; Mehanna et al., 2023). HPV status was not correlated to the expression of examined fibroblast markers. These results contrast a previous report by Wang et al. (2022) who reported lower α-SMA expression in HPV-positive HNSCC compared to HPV-negative tumors. This discordance may be explained by the method used for HPV detection. While Wang et al. based HPV status solely on p16 expression, we used combined positivity of HPV DNA and p16 to define HPV-positive tumors as recent evidence suggests this classification to be a better predictor of the improved survival rates conferred by HPV-positive tumors (Mehanna et al., 2023). Stratification of HPV-positive tumors according to FAP, PDGFRb, periostin and α-SMA did not reveal significant association with overall survival in this subgroup (Supplementary Figure S2). Nonetheless, future studies should further examine HPV status in relation to specific CAF subgroups, as HPV serves as a crucial distinguishing characteristic in OPSCC.

Recent studies investigated possible subtypes of CAFs, categorizing them according to characteristic marker genes into inflammatory CAFs (iCAF), myo-cancer-associated fibroblasts (mCAF) and antigen presenting CAFs (apCAFs) (Elyada et al., 2019; Zhang et al., 2021; Wang et al., 2022). The role of intratumoral MHCII restricted antigen presentation by apCAFs remains elusive. While apCAFs isolated from orthotopic mouse tumor models were shown to induce lymphocyte activation, as measured by CD25 and CD69 upregulation, in an antigen specific manner, apCAFs express low levels of costimulatory molecules CD80, CD86 and CD40 when compared to professional antigen presenting cells (Elyada et al., 2019). A similar subset of apCAFs was detected in single cell RNA-seq of HNSCCs (Zhang et al., 2021). Expression of fibroblast markers FAP, PDGFRb and α-SMA is highest in mCAFs but PDGFRb and FAP gene expression was detectable in other CAF subsets as well (Elyada et al., 2019; Zhang et al., 2021). We included CD8 T-cell infiltration in our analysis to elucidate intratumoral cytotoxic lymphocyte abundance in the context of CAF background. In line with previous reports from HPV-positive and negative OPSCC, high CD8 T-cell infiltration facilitated improved patient survival (Jung et al., 2013; Haist et al., 2023). Furthermore, CD8 infiltration was positively associated with FAP and PDGFRb high tumors. CAFs can modulate intratumoral immune cell abundance via cytokines, growth factors and chemokines including CC-chemokine 2 (CCL2), CCL5, colony-stimulating factor 1 (CSF1), CXC-chemokine 5 (CXCL5), CXCL9 and CXCL10 (Chen et al., 2021). Grout et al. and others established a pattern of CAF mediated T-cell exclusion which is associated with resistance to immunotherapy (Ford et al., 2020; Grout et al., 2022). High levels of intratumoral periostin have been associated with increased infiltration of PD-L1 positive, immunosuppressive M2-like macrophages, thus the dismal overall survival of CD8^low^ periostin^high^ patients may be linked to high levels of M2-like macrophage mediated inhibition of effector T-cells (Wei et al., 2023). Within the CD8^high^ population, PDGFRb^high^ status delineates patients with impaired survival. Previous reports describe elevated evasion of immune surveillance mediated by intratumoral transforming growth factor-beta (TGF-β) signaling, which is positively associated to PDGFRb expression (Baba et al., 2022; Zhang Y. et al., 2022). In an analysis of metastatic urothelial cancer, a high TGF- β signature in fibroblasts attenuated response to PD-L1 blockade and contributed to CD8 T-cell exclusion (Mariathasan et al., 2018). Further analyses of the tumor stroma and invasive margin are warranted to specifically address differential heterogenous CAF populations in the context of tumor immune infiltration, patient survival and sex specific aspects in OPSCC.

These analyses may encompass a thorough exploration and enhanced comprehension of additional CAF markers, notably integrins. Integrins are recognized for their interactions with growth factors and exhibit co-expression patterns with various other CAF markers. Notably, the overexpression of Integrin α11 in head and neck squamous cell carcinoma demonstrated a positive correlation with α-SMA, underscoring the intricate interplay among these markers in the context of tumor biology (Parajuli et al., 2017).

An intriguing avenue for additional research could delve into the interaction of another PDGF receptor isoform, namely, PDGFRα. While both PDGFR isoforms exhibit some overlap in signaling pathways, there is compelling evidence pointing to a distinctly diverse impact on fibroblasts. This includes instances where PDGFR, depending on isoform, demonstrates the ability to either inhibit or stimulate fibroblast chemotaxis (Heldin et al., 1985; Heldin and Westermark, 1999). Exploring the co-expression patterns of PDGFRα with the markers discussed here could provide valuable insights, shedding light on the intricate interactions among CAF markers.

A limitation of the IHC analyses described here is the lack of an accepted cutoff for positivity of fibroblast markers. After careful evaluation and review of the literature we chose to use the median expression in our patient population as there is no established consensus on cutoffs and reported thresholds vary broadly (Tsujino et al., 2007; Liu et al., 2016; Fang et al., 2022). Previous publications from our group and others successfully used a similar classification to analyze CAFs in other tumor entities (Costa et al., 2018; Knipper et al., 2023). Investigating the association of treatment response and CAFs is a topic of great importance to allow patient selection based on CAF marker expression. Unfortunately, a rigorous investigation of response rates to different treatment modalities in relation to CAF marker expression was not feasible in the setting of this study, as no sufficient data regarding adjuvant are available to the authors and missing data points would have introduced a substantial bias.

Given the expanding complexity of differential fibroblast sets in cancers, the present study enhances the current understanding of CAFs in OPSCC. Importantly, we establish PDGFRb^low^ α-SMA^low^ periostin^low^ status as an independent predictor of improved survival. Future studies exploring the reciprocal shaping of cancer cells, fibroblasts and tumor infiltrating immune cells are warranted and should consider multiplexed analyses to allow the specific classification of CAF subsets.

## Data Availability

The raw data supporting the conclusion of this article will be made available by the authors, without undue reservation.

## Abbreviations

OPSCC, oropharyngeal squamous cell carcinoma; CAF, carcinoma associated fibroblast; FAP, fibroblast activation protein; PDGFRb, platelet-derived growth factor receptor beta; α-SMA, alpha smooth muscle actin; HNSCC, head and neck squamous cell carcinoma; HPV, human papilloma virus; ICB, immune checkpoint blockade; TME, tumor micro environment; ECM, extracellular matrix; CAR T-cells, chimeric antigen receptor T-cells; FAPI, fibroblast activation protein inhibitor; PET, positron emission tomography; myCAF, myofibroblasts; ICD-O, International Classification of Diseases for Oncology; TMA, tissue microarrays; FFPE, Formalin fixed paraffin embedded; p16^INK4a^, p16; IHC, immunohistochemistry; OS, overall survival; HR, hazard ratio; CI, confidence interval; G, grade; iCAF, inflammatory carcinoma associated fibroblast; mCAF, myo carcinoma -associated fibroblast; apCAF, antigen presenting carcinoma associated fibroblast; TGF-β, transforming growth factor-beta; CCL2, CC-chemokine 2; CSF1, colony-stimulating factor 1; CXCL5, CXC-chemokine 5.
