# Supplementary material for: Co-expression patterns of cancer associated fibroblast markers reveal distinct subgroups related to patient survival in oropharyngeal squamous cell carcinoma
Source: Front Cell Dev Biol. 2024 Jan 24;12:1337361. doi: 10.3389/fcell.2024.1337361 (PMC10847231; doi:10.3389/fcell.2024.1337361)
Supplement: Supplementary file 1 [file Table1.docx]

| Antibody | Manufacturer | Clone | Dilution | Pretreatment | Control | Positive | Negative | Order |
| --- | --- | --- | --- | --- | --- | --- | --- | --- |
|  |  |  |  |  | tissue | control | control | number |
| α-SMA | Dako | 1A4, mouse | 1:4000 | w/o | A | Smooth muscle cells | Epithelial cells | M0851 |
| FAP | Abcam | EPR20021 | 1:200 | Citrat | Colon, Mamma | Stromal fibroblasts | Cancer cells | Ab207178 |
| PDGFRb | Abcam | Y92, rabbit | 1:300 | EDTA | Prostate | Stromal cells | Cancer cells | Ab32570 |
| Periostin | Abcam | EPR19934 | 1:2000 | EDTA | Colon | Extracellular matrix | Epithelial cells | Ab219056 |
| p16 | Zytomed | JC2, mouse | 1:100 | EDTA | p16/WT1 pos. OC | Tumor cells |  | MSK123-05 |
| CD8 | Dako | C8/144B, mouse | 1:200 | Citrat | Tonsil | T-Lymphocytes | Epithelial cells | M7103 |

**Supplementary Table S1** Detailed antibody information; A: appendix vermiformis, OC: Ovarial carcinoma
