# Supplementary material for: Co-expression patterns of cancer associated fibroblast markers reveal distinct subgroups related to patient survival in oropharyngeal squamous cell carcinoma
Source: Front Cell Dev Biol. 2024 Jan 24;12:1337361. doi: 10.3389/fcell.2024.1337361 (PMC10847231; doi:10.3389/fcell.2024.1337361)
Supplement: Supplementary file 2 [file DataSheet1.docx]

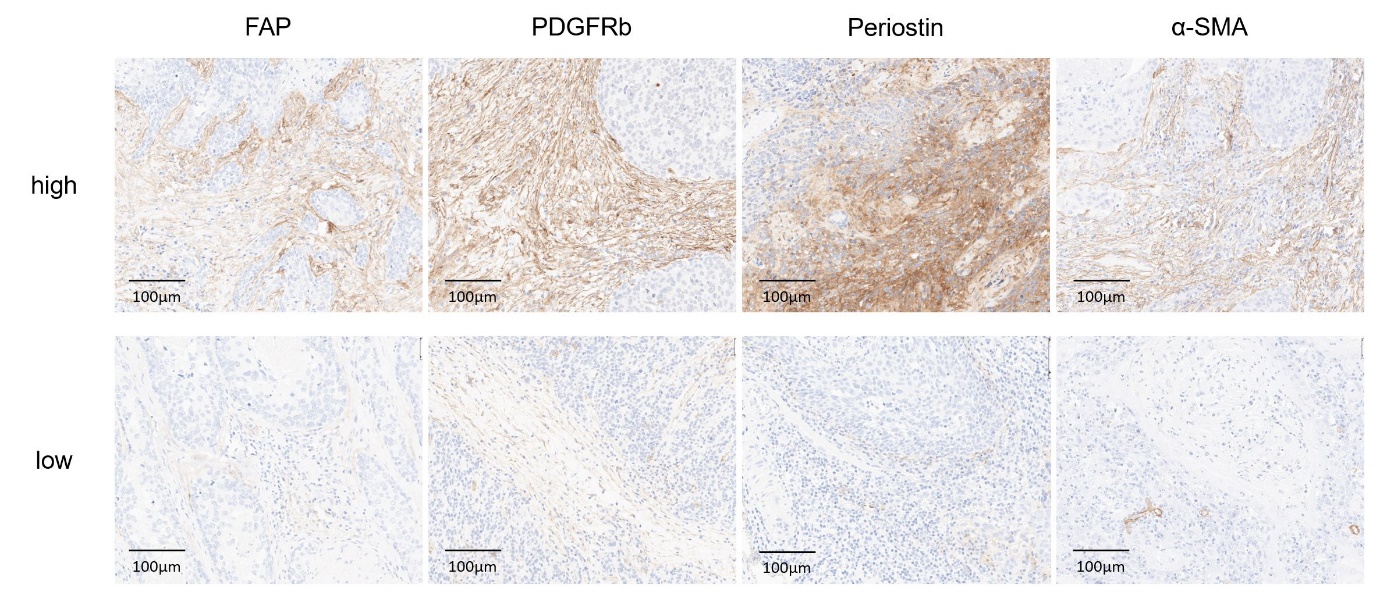


**Supplementary Figure S1** Representative images of immunohistochemical stainings (top high, bottom low) for fibroblast activation protein (FAP), platelet derived growth factor receptor beta (PDGFRb), periostin and α-smooth muscle actin (α-SMA)


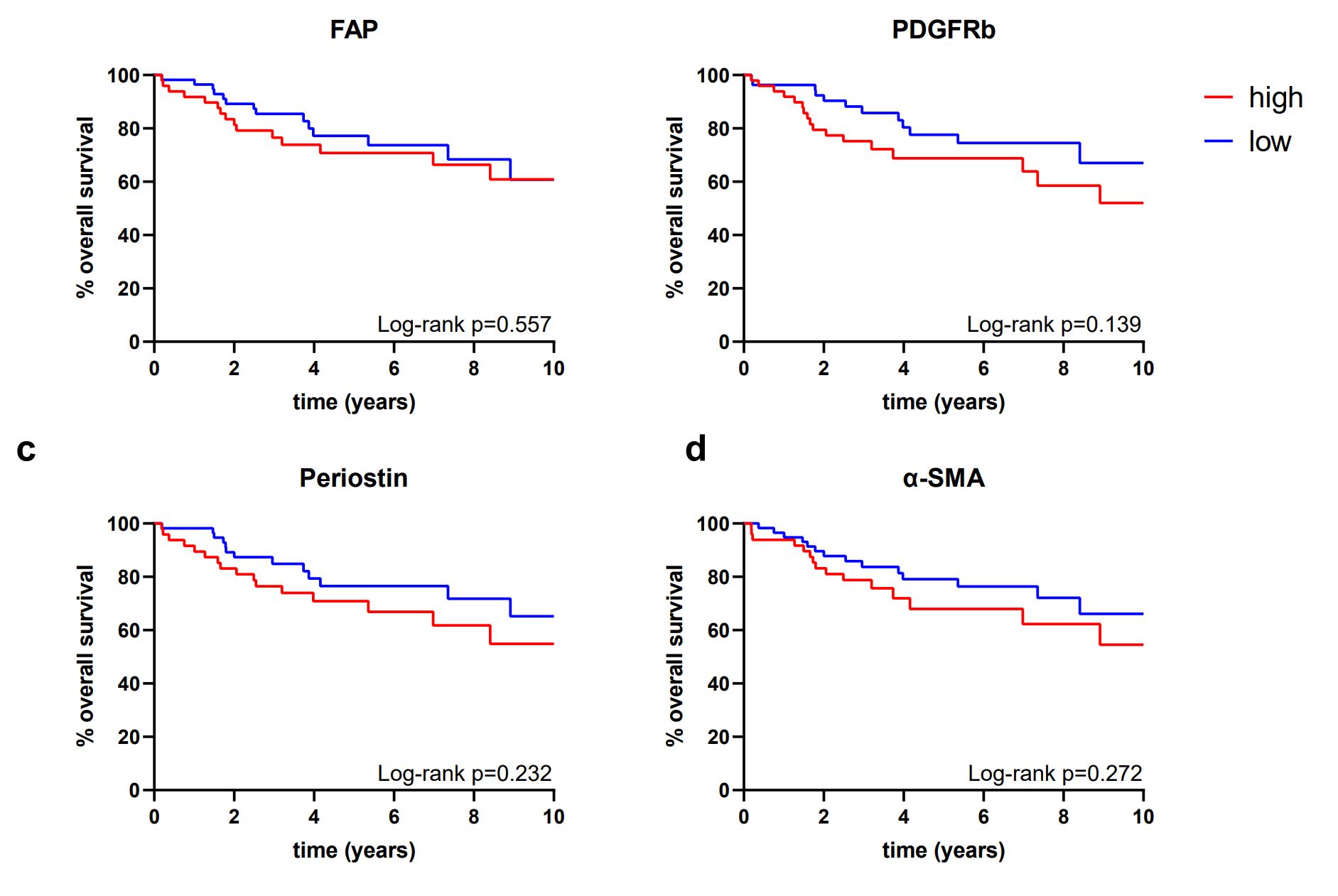


**Supplementary Figure S2** Kaplan-Meier curves for overall survival of human papilloma virus (HPV)-positive tumors according to (a) fibroblast activation protein (FAP) (high n=49, low n=57), (b) platelet derived growth factor receptor beta (PDGFRb) (high n=49, low n= 54), (c) periostin (high n=48, low n=57) and (d) α-smooth muscle actin (α-SMA) (high n= 49, low n=58) expression; *p*-values calculated by log-rank test
